# Supplementary material for: A set of multi-entry identification keys to African frugivorous flies (Diptera, Tephritidae)
Source: Zookeys. 2014 Jul 24;(428):97–108. doi: 10.3897/zookeys.428.7366 (PMC4143993; doi:10.3897/zookeys.428.7366)
Supplement: Supplementary material 6 — Key to Ceratitis [file zookeys-428-097-s006.zip › SF6_ZooKeys_key to Ceratitis/key/SF6_ZooKeys_key to Ceratitis/Media/Html/Ceratitis hancocki .htm]

Ceratitis (Ceratalaspis) hancocki sp


***Ceratitis*** **(*Ceratalaspis*)
*hancocki* De Meyer**

*Ceratitis* (*Ceratalaspis*)
*hancocki* De Meyer, 1998: 279.

Body
length: 4.64 (4.60-4.70) mm; wing length: 4.59 (4.40-4.85) mm.

Male

Head.
Antenna yellow-orange. Third antennal segment slightly less than three times as
long as second segment. Arista with short to medium long hairs over entire
length. Frons yellow; with short scattered hairs which are distinctly darker
than the frons; in lateral view not distinctly projecting forwards at antennal
implant. Chaetotaxy normal for subgenus.

Thorax.
Postpronotum yellow, almost same colour as centre of mesonotum; postpronotum
spotted but spot small. Ground colour of mesonotum pale with yellow-orange
tinge. Chaetotaxy normal for subgenus. Scapular setae black, inner reddish. One
anepisternal bristle. Scutellum mainly yellow; with three black separate
markings apically except at ventral margin where narrowly joining; basally two
dark distinct spots. Subscutellum pale with three dark separate spots except
along dorsal edge where touching.

Legs
yellow; setation typical for subgenus, mainly pale especially on femora.
Posterior and posterodorsal rows on fore femur pale. Ventral spines on fore
femur reddish.

Wing
bands with markings yellow-brown. Banding as in fig. 1b, marginal band continuous;
cubital band joined with discal band; medial band absent; crossvein r-m at
middle of discal cell. Crossvein dm-cu posteriorly more inwards than
anteriorly.

Abdomen.
Yellow. Setation and banding typical for subgenus.

Female

As
male except for the following� characters: postpronotal spot sometimes
obscured. Scapular setae and ventral spines darker. Mesonotum with postsutural
median spot absent. Oviscape shorter than abdominal terga 3-6 combined.

(Description
after De Meyer, 1998)
